# Effectiveness of fullerene/magnesium oxide nanocomposite in removing ciprofloxacin and tetracycline from aqueous solutions†

**DOI:** 10.1039/d4ra07938h

**Published:** 2025-02-17

**Authors:** Sammer M. Bekhit, Sahar A. Zaki, Mohamed Salah El-Din Hassouna, Marwa Elkady

**Affiliations:** a Institute of Graduate Studies and Research, Alexandria University Alexandria 21526 Egypt; b Environmental Biotechnology Department, Genetic Engineering and Biotechnology Research Institute (GEBRI), City of Scientific Research and Technological Applications (SRTA-City) Alexandria 21934 Egypt; c Chemical and Petrochemical Engineering Department, Egypt-Japan University of Science and Technology (E-JUST) New Borg El-Arab City Alexandria 21934 Egypt; d Fabrication Technologies Researches Department, Advanced Technology and New Materials Research Institute, City of Scientific Research and Technological Applications (SRTA-City) Alexandria 21934 Egypt Igsr.sammer@alexu.edu.eg

## Abstract

The excessive use of antibiotics, including ciprofloxacin (CIP) and tetracycline (TC), poses negative impacts on both human health and ecosystems. In this work, fullerene/magnesium oxide (F/MgO) nanocomposite was prepared and studied as adsorbent for CIP and TC removal. Adding metal oxide to F led to a change in its characteristics which was confirmed by XRD, FTIR, SEM, and TEM. A maximal removal for 50 mg L^−1^ CIP was 84.6% at 60 min, pH 7, and 0.2 g L^−1^ of adsorbent dose. 43.6% of 50 mg L^−1^ of TC adsorbed at 60 min, pH 5, and 1 g L^−1^ of adsorbent dose. Adsorption thermodynamics elucidated that the adsorption on F/MgO nanocomposite were spontaneous and exothermic, and non-spontaneous and endothermic for CIP and TC, respectively. Pseudo-second-order kinetic model fitted well the adsorption data of CIP and TC. Various coexisting ions had different impacts on the adsorption efficiency of CIP and TC. The competitive adsorption between CIP and TC on the surface of F/MgO nanocomposite was studied. The F/MgO nanocomposite was efficiently reused 5 cycles for CIP and TC removal and remained effective. This work explores a novel adsorbent for the elimination of CIP and TC from aqueous solutions.

## Abstract 2

Effective removal of ciprofloxacin and tetracycline using fullerene/magnesium oxide nanocomposite.

## Graphical abstract

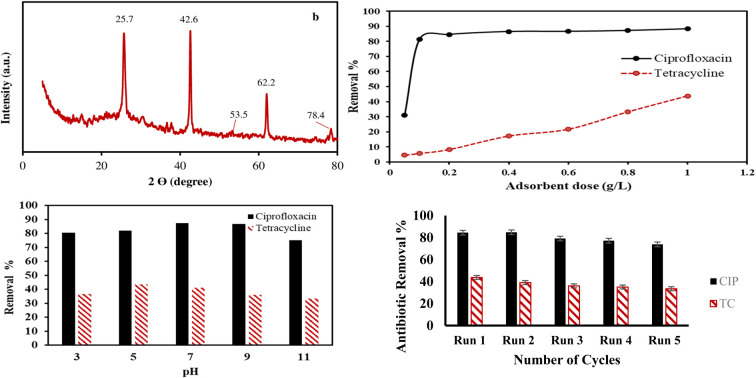

## 1. Introduction

The quality of water bodies influences environmental safety and public health.^1^ The presence of pharmaceutical compounds in aquatic bodies has negative impacts on the ecosystem.^2–4^ Antibiotics are considered prevalent contaminants of pharmaceutical compounds. Presently, high usage of antibiotics and their discharge from pharmaceutical industries, hospitals, human waste, and veterinary fields led to contamination and increase bacterial resistance.^5^ The human consumption of antibiotics, represented in defined daily doses, will reach to 42 billion in 2030. The demand of antibiotics in livestock is anticipated to increase to 104 079 tons in 2030.^6^

Among different antibiotics, ciprofloxacin (CIP) and tetracycline (TC) are the most prevalent antibiotics globally because of their high efficacy and low cost. CIP and TC are commonly prescribed for treating human and animal diseases caused by fungi, bacteria, parasites, and rickettsia. Furthermore, they are utilized in livestock breeding to treat and prevent diseases, and are used as feed additives in poultry and aquaculture.^5,7^

Only 20–50% of the antibiotics can be degraded in human and animal bodies, the residual antibiotics are excreted without being metabolized because they have hydrophilic characteristics and low biodegradability.^7^ The presence of residual antibiotics in the environment poses a threat to the organisms in the ecosystem. Moreover, it increases the production of resistance genes and resistant microbes against antibiotics.^7–9^ Antimicrobial resistance is estimated to cause about 10 million deaths and financial losses exceeding 100 trillion dollars by 2050.^10^

Therefore, treatment of residual antibiotics is required before discharging into water surfaces. Different methods are used for the elimination of antibiotics such as photocatalysis,^11^ ozonation,^12^ coagulation,^13^ liquid membrane separation,^14^ and adsorption.^15^ Among these treatment methods, adsorption has been preferred for removing antibiotics because of its high efficiency, simplicity in design, absence of toxic byproducts, and relatively lower cost.^16–18^ An adsorbent material should be chemically and physically stable, nontoxic, and recyclable.^4^ The adsorption capacity is affected by the porosity, surface area, surface charge, and pore diameter of the adsorbent.^7,8^

Nanomaterials are preferred for removing water pollutants.^19^ MgO nanoparticles have gained significant attention in wastewater treatment because of various advantages, such as ease of production, non-toxicity, large surface area, and high porosity.^20,21^ Moreover, MgO nanoparticles have more economic benefits compared to other adsorbents.^22^ MgO nanoparticles could have different morphological structures as flowers, cubes, platelets, spheres, needles, rods, and stars, which are characterized by various novel properties.^21^

Carbon is one of the most abundant elements on Earth.^23^ Carbon-based nanomaterials, including fullerenes, carbon nanotubes, and porous carbons, are considered effective adsorbents because of their low toxicity, high chemical stability, and high adsorption capacity. Moreover, many researchers investigated the functionalized C_60_ fullerenes in their studies because the chemical reactivity and absorption capacity of functionalized fullerenes can be increased towards the target pollutant.^24,25^

Recently, fullerene (F) has been combined with a metal oxide to form a composite, which is used for pollutant removal. However, the application of F/MgO nanocomposite as an adsorbent for antibiotic removal has not been explored. Accordingly, our study aims to determine the removal efficiency of CIP and TC using F/MgO nanocomposite as an adsorbent. MgO was synthesized by the chemical precipitation method. F/MgO nanocomposite was prepared by ball milling and characterized by X-ray Diffraction (XRD), Fourier transform infrared (FTIR), scanning electron microscopy (SEM), and transmission electron microscopy (TEM). The adsorption behavior of CIP and TC on F/MgO nanocomposite was investigated by optimizing various factors, including contact time, initial pH, doses of adsorbent; initial antibiotic concentrations, temperature; and presence of coexisting ions. Thermodynamic and kinetic models were also determined. Moreover, the effect of a binary system consisting of both CIP and TC on their adsorption removal was studied. Environmental applications for CIP and TC removal using F/MgO nanocomposite were investigated.

## 2. Materials and methods

### 2.1. Chemicals

Tetracycline hydrochloride (ultra-pure) was obtained from Bio Basic INC., Canada and ciprofloxacin (98% purity) was brought from Acros Organics, USA. Fullerene (7–12 nm OD, 0.5–10 micron long) was purchased from Alfa Aesar, USA. Magnesium chloride anhydrous (MgCl_2_) was received from Daejung CO., South Korea. All chemicals were of analytical grade which used directly without any additional purification. HCl (1 M) and NaOH (1 M) solutions were prepared to manage pH of the antibiotic solutions.

### 2.2. Preparation of F/MgO nanocomposite

MgO nanoflowers were prepared by the chemical precipitation method and characterized as reported in our recent publication.^26^ The preparation of MgO is provided in Text 1 (ESI†). In this study, F/MgO nanocomposite was prepared by ball milling (Photon Scientific, Egypt) that avoid the usage of toxic solvents or costly purification processes. Equal amounts of F and MgO were milled in agate jar using agate balls with a milling speed of 600 round per minute for 12 h at room temperature.

### 2.3. Characterization of the adsorbent material

The crystalline phase of F/MgO nanocomposite was determined by using XRD (Schimadzu-7000, Japan). The Debye–Scherrer formula was used to calculate the average crystallite size of the adsorbent (*D*)^27^
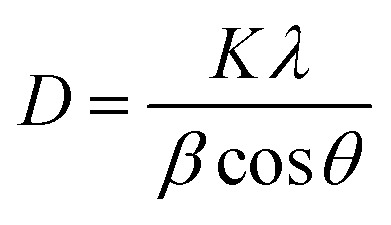
where *K* (0.94) is the Scherrer factor, *λ* (1.54178 Å) is the wavelength of X-ray radiation. *β* is full width at half maximum of XRD peak, *θ* is the reflection angle.

The surface area of F/MgO nanocomposite was obtained at 77.3 K by the Brunauer–Emmett–Teller (BET) method (BEL Japan, Inc., Version 6.3.0.0). FTIR spectroscopy (Spectrum BX 11-LX 18-5255 PerkinElmer, USA) was applied to determine the functional groups of the nanocomposite. The morphological characteristics of adsorbent were examined by SEM (JEOL, JSM-6360, Japan) and TEM (JEOL JEM-1230, Japan).

### 2.4 Adsorption experiments

All experiments for CIP and TC in this study were carried out in batch mode under continuous stirring at 150 rpm in darkness. After adsorption process, the samples were centrifuged at 6000 rpm for 5 min, and the supernatants of CIP and TC were analyzed by UV/VIS spectrophotometer (Evolution 300™, Thermo Scientific™, USA) at 278 and 358 nm, respectively. The antibiotic removal percentage was calculated by the following equation
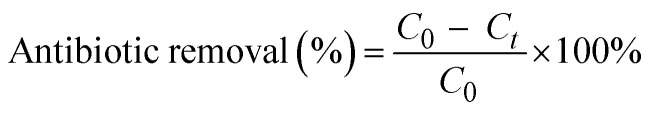
where *C*_0_ is the initial concentration of antibiotic at the beginning of the experiment and *C*_*t*_ is antibiotic concentration of the sample after the *t* time in minutes.

The effects of various experimental conditions on the removal of 50 mg L^−1^ of CIP and TC (40 mL) were studied, such as contact time (0–120 min), initial pH (3, 5, 7, 9, and 11), adsorbent dose (0.1–1 g L^−1^), initial antibiotic concentration (10, 20, 30, 40, 50 and 60 mg L^−1^), and temperature (30, 40 and 50 °C) at 150 rpm. The adsorption processes of antibiotics were identified by UV/VIS spectrophotometer and FTIR.

After optimizing the previous parameters, different concentrations of Na^1+^ (0.01–0.1 M), Ca^2+^ (0.01–0.1 M), and 0.01 M of various co-existing cations as K^1+^, Mg^2+^, anions as NO_3_^1−^, SO_4_^2−^, H_2_PO_4_^1−^, as well as some metals, Fe^3+^, Zn^2+^, Cu^2+^, were investigated to determine their potential impacts on antibiotic removal efficiency. The adsorption selectivity of the nanocomposite between CIP and TC was investigated by adding different concentrations of TC or CIP (5–20 mg L^−1^) to 50 mg L^−1^ CIP or TC.

### 2.5. Removal of CIP and TC from different water sources

Two samples of water were used to determine the efficiency of F/MgO nanocomposite in CIP and TC removal. The samples were tap water and raw water (obtained from a drinking water canal, Alexandria, Egypt). The raw water was filtered to remove any suspended matter. CIP and TC were not detected in these samples. 50 mg of antibiotic was added to 1 L of those samples to investigate the removal efficiency of the adsorbent under the optimized conditions.

### 2.6 Reusability

Reutilizing of the adsorbent was tested to check any decrease in CIP and TC removal. Therefore, the adsorbent was regenerated and reused five times to determine its efficiency at the optimized conditions in the adsorption processes. After each test, the adsorbent was separated, sonicated with a mixture of 3% methanol (16 mL) and 3% NaOH (4 mL) for 5 min. Then, the adsorbent was centrifuged, washed three times, dried at 110 °C for 1 h, and reused.

## 3. Results and discussions

### 3.1 Characterizations of the adsorbent

**Fig. 1:**
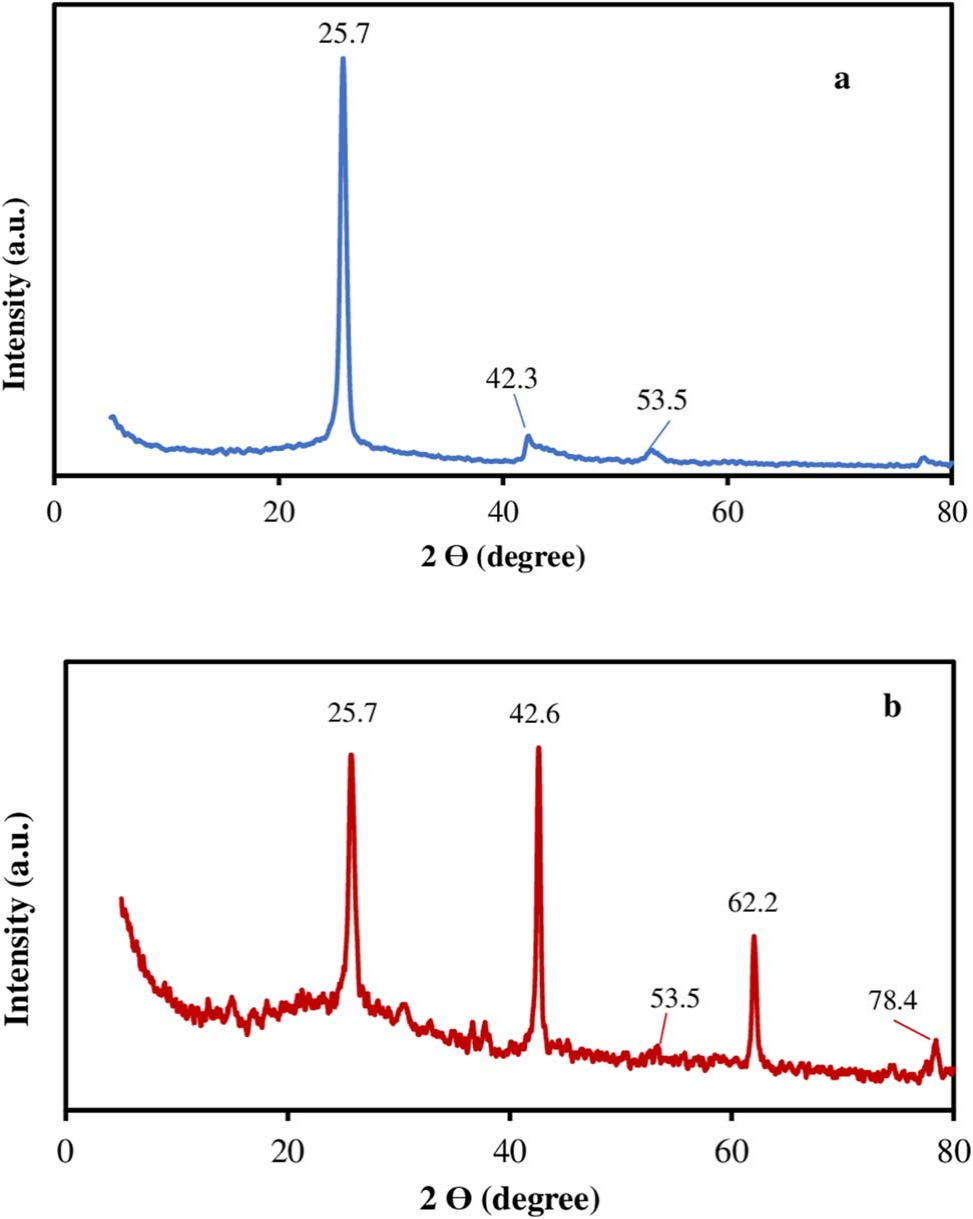
XRD of F (a) and F/MgO nanocomposite (b).

XRD patterns of F and its nanocomposite are presented in Fig. 1. The diffraction peaks at 2*θ* values (Fig. 1a) of 25.7°, 42.3°, and 53.5° belong to F that correspond to (002), (101), and (004) planes, respectively. These findings are similar to those reported at ICDD card No. 00-056-0159.^24^ The appearance of characteristic peaks at 25.7°, 42.6° and 53.5° in Fig. 1b proves the presence of F in the F/MgO nanocomposite. According to JCPDS file No. 01-089-7746,^20,26^ the characteristic diffraction peaks at 2*θ* values of 42.6°, 62.2°, and 78.4°, attributed to (200), (220), (222) planes, respectively, confirms the presence of MgO in the nanocomposite, as shown in Fig. 1b. It is observed that the peak positions of MgO and F do not change in Fig. 1b. Accordingly, it is concluded that the addition of MgO does not change the crystallinity of F. The average crystalline size of the F/MgO nanocomposite was 24.94 nm which was calculated using the Debye–Scherrer formula.

**Fig. 2:**
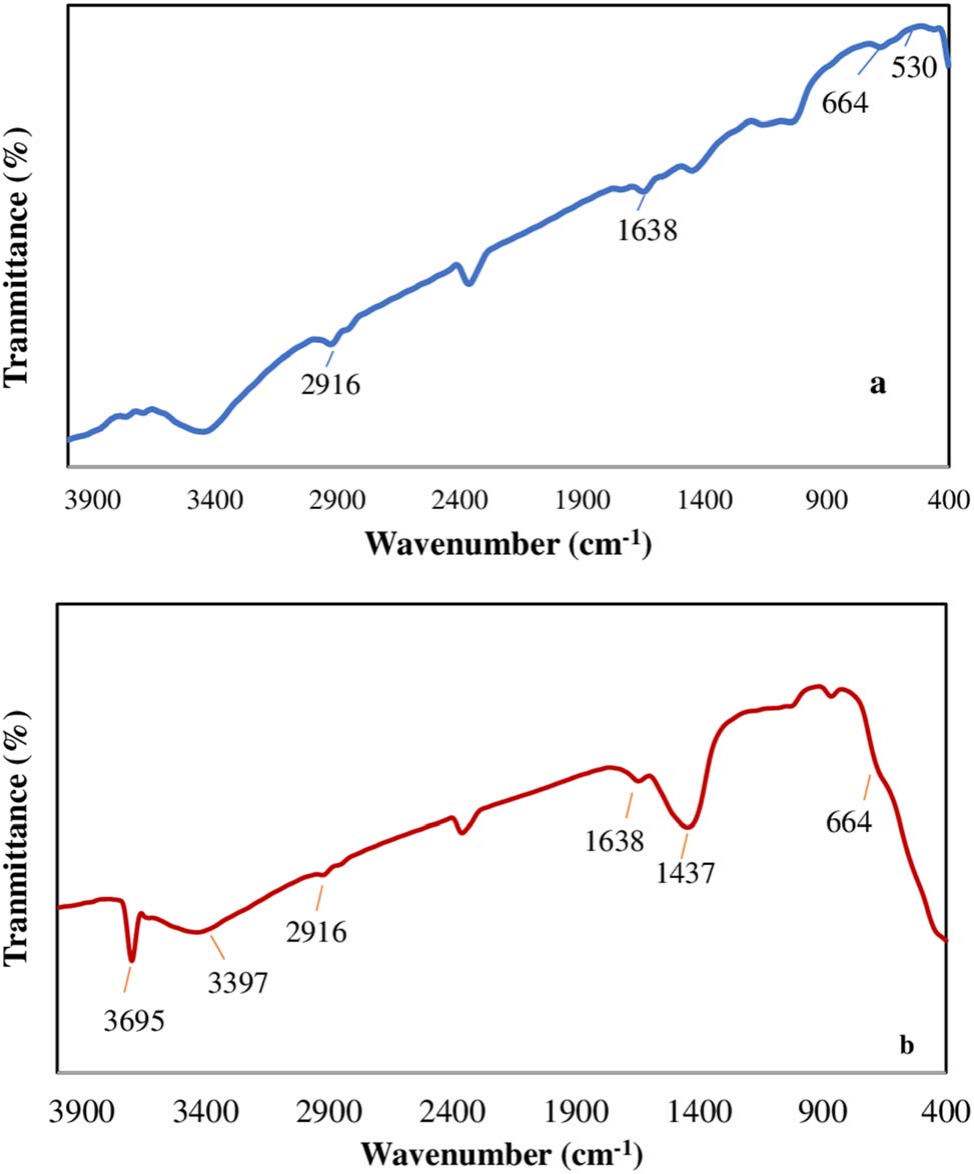
FTIR of F (a), F/MgO (b).

In order to investigate the functional groups of F, and F/MgO nanocomposite, FTIR spectroscopy was utilized in the wave number range of 4000–400 cm^−1^ and the results are shown in Fig. 2. The characteristic absorption spectrum of F nanoparticles in Fig. 2a shows that the peak at 2916 cm^−1^ refers to stretching vibrations of CH_2_. Furthermore, the (C=C) stretching vibration of the C60 cage can also be observed at 1638 cm^−1^. Moreover, the two peaks at 664 cm^−1^ and 530 cm^−1^ resulting from a primarily radial motion of the carbon atoms.^28,29^

Fig. 2b presents the IR spectrum of the F/MgO nanocomposite. The spectrum depicts the similar bands of F (2916, 1638, 664 cm^−1^), which confirm the presence of F in the nanocomposite. The bands of 3695, 3397 and 1437 cm^−1^ bands correspond to MgO bands. A sharp peak at 3695 cm^−1^ assigned to stretching mode of the free hydroxyl group (–OH). Moreover, the broad peak at 3397 cm^−1^ corresponded to the OH stretching vibrations of adsorbed water molecules on the MgO NFs surface. The peak at 1437 cm^−1^ is ascribed to the Mg–O stretching vibrations as well as the Mg–O–Mg deformation vibrations. The FTIR spectrum of MgO NFs has been mentioned in our previous study.^26^

**Fig. 3:**
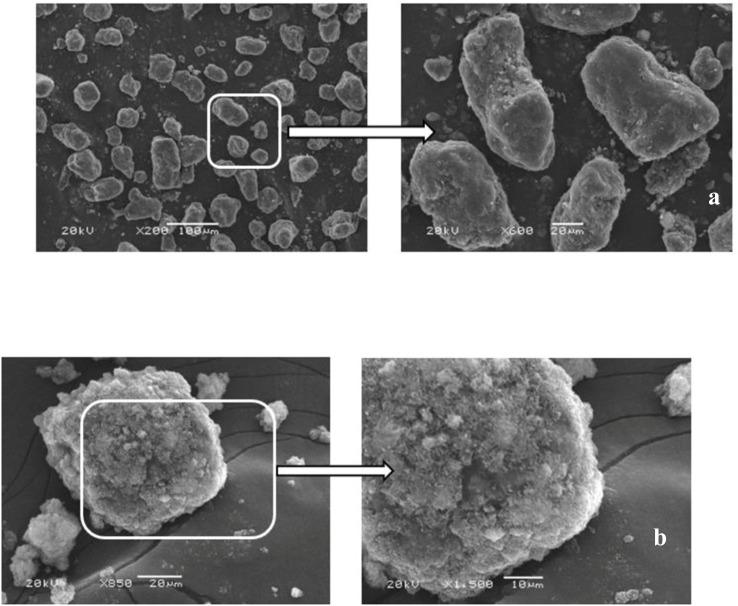
SEM images of F (a), F/MgO nanocomposite (b).

**Fig. 4:**
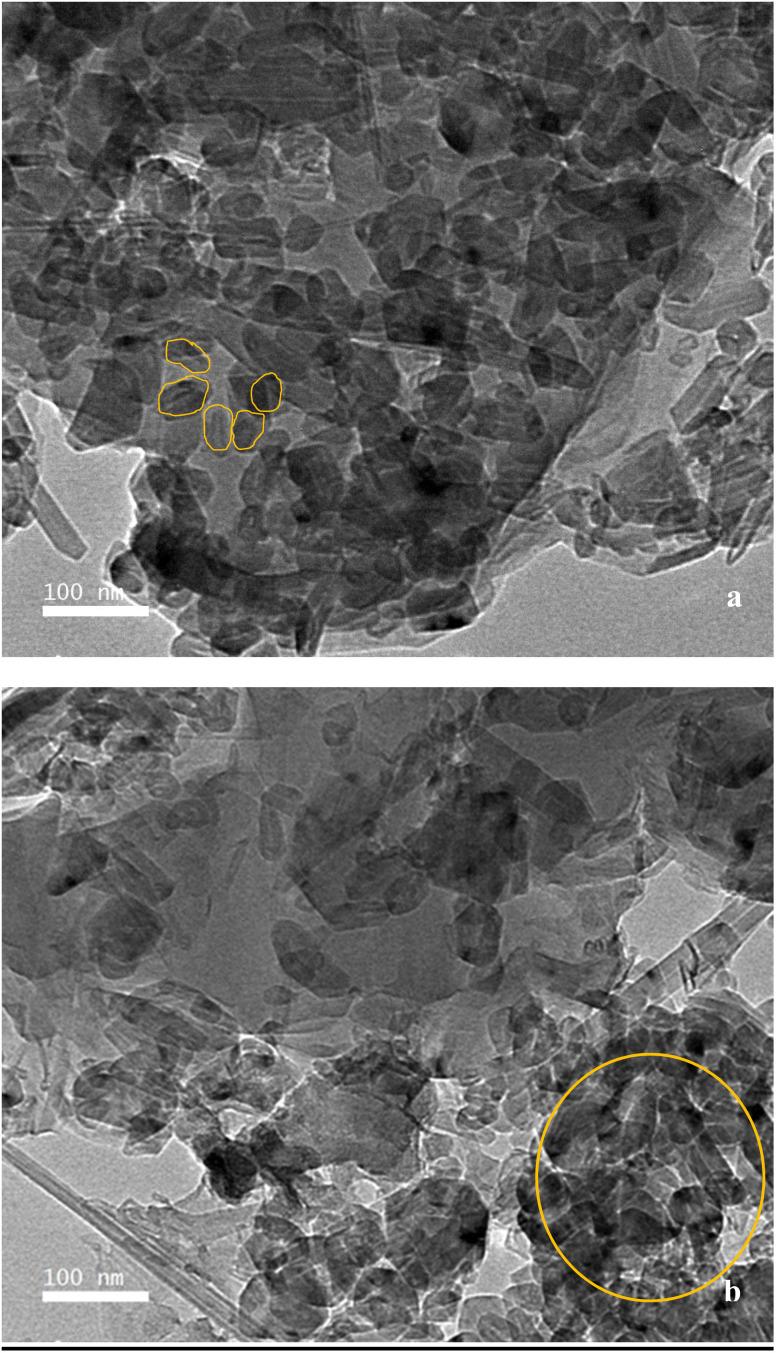
TEM images of F (a), F/MgO nanocomposite (b).

The morphology of F and F/MgO nanocomposite were examined using SEM (Fig. 3) and TEM (Fig. 4). F has amorphous granular particles, as presented in Fig. 3a. The surface of the nanocomposite is significantly different from F. As shown in Fig. 3b, MgO show agglomerated flower-like spheres on the surface of F. The specific surface area of F/MgO nanocomposite was calculated as 111 m^2^ g^−1^ using BET analysis. Nanoflowers exhibit a high surface-to-volume ratio that enhance surface adsorption and accelerate reaction kinetics.^30^ The TEM image presents F with close sheets of amorphous shapes (Fig. 4a). Fig. 4b confirms agglomerations between F and MgO in the F/MgO nanocomposite.

### 3.2 Antibiotic removal using different adsorbents

The removal of CIP and TC were investigated using various adsorbents. The removal performance of F/MgO nanocomposite was evaluated comparing to its single components (pure F and MgO). The removal (*R*%) and adsorption capacity (*q*_e_) of CIP and TC is summarized in Table 1. The adsorbent dosage was 1 g L^−1^ for removal 50 mg L^−1^ of each antibiotic without pH control for 60 min at 30 °C. The *R*% and *q*_e_ of CIP and TC using F/MgO nanocomposite were higher than using pure F or MgO, as shown in Table 1. The highest removal percentage (87.2 & 42.0%) and adsorption capacity (21.0 & 43.6 mg g^−1^) using F/MgO nanocomposite for CIP and TC, respectively, were ascribed to the synergistic effect of F and MgO that increase the accessible binding sites on the surface of the adsorbent.

**Table 1:** Removal percentage and adsorption capacity of CIP and TC using different adsorbents

Adsorbent	*R*%	*q*_e_ (mg g^−1^)
**For CIP**		
F	9.4	23.5
MgO	43.8	109.5
F/MgO	87.2	218.0

**For TC**		
F	8.2	4.1
MgO	35.0	17.5
F/MgO	42.00	21.0

### 3.3 Effect of various parameters on CIP and TC removal using F/MgO nanocomposite

#### 3.3.1 Effect of contact time

**Fig. 5:**
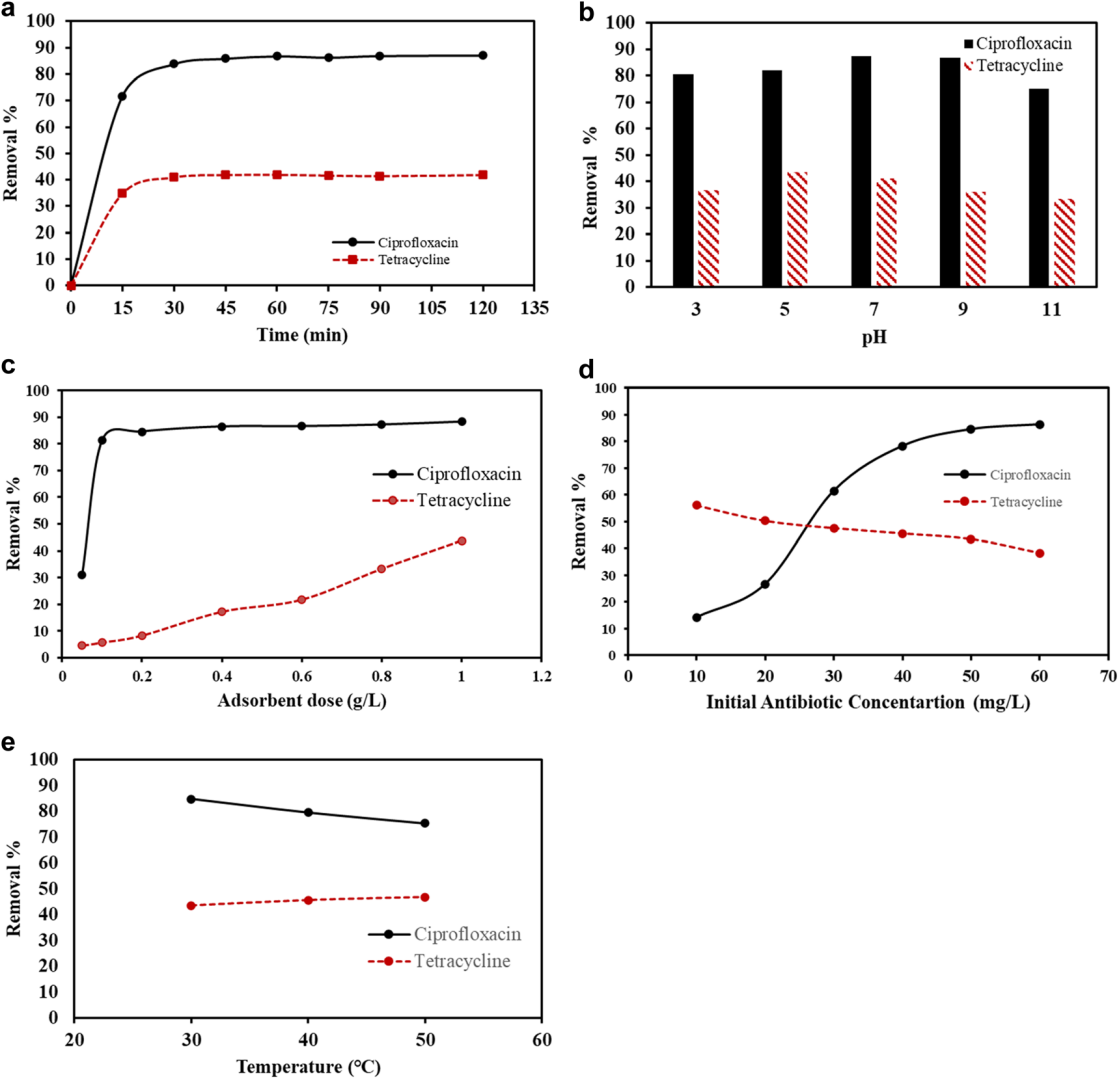
(a) Effect of time on CIP and TC removal. (b) Effect of pH on CIP and TC removal. (c) Effect of dose on CIP and TC removal. (d) Effect of initial antibiotic concentration on CIP and TC removal. (e) effect of temperature on CIP and TC removal.

Adsorbent dose of 1 g L^−1^ was tested for removing 50 mg L^−1^ of antibiotics. As shown in Fig. 5a, it is evident that CIP and TC removal increased rapidly at the initial 15 min, the removal for CIP and TC were 72% and 35%, respectively, which could be due to presence of many readily accessible sites for adsorption. Then, the removal of both antibiotics increased slowly due to a continuous reduction in the number of active sites of the adsorbent. The removal became nearly constant in both curves after 30 min of contact time illustrating that the F/MgO nanocomposite was saturated at this level. The maximum removal percentages for CIP and TC were 87% and 42%, respectively, which were obtained after 60 min of the adsorption process. Therefore, 60 min of contact time was used in all next experiments. The explanations of our study are in agreement with earlier reports.^2,19,31^

#### 3.3.2 Effect of pH

Initial pH of the solution is considered a critical factor in influencing the adsorption process between the adsorbent and adsorbate; since the surface charge of the adsorbent and the ionization states of the organic molecules could be modified by changing pH of the solution.^20,32^ To investigate the effect of pH on the adsorption behavior, a batch sorption experiment was studied at different pHs (3–11) with the initial antibiotic concentration of 50 mg L^−1^ using 1 g L^−1^ of F/MgO nanocomposite for 60 min at 30 °C.

The point of zero charge (PZC) of F/MgO nanocomposite is around 10 (Fig. S1†), it is the pH value when the surface charge of the adsorbent is neutral. The surface of the adsorbent will be a negatively charged surface at pH > PZC and a positively charged surface at pH < PZC. The adsorption behavior can be considered based on the electrostatic interactions between the surface charge of the adsorbent and the charge of antibiotic molecules.^14^

The CIP molecule is basically an amphoteric organic molecule due to the existence of a carboxylic acid group and a secondary amine group with p*K*_a_ of 5.9 and 8.9, respectively. Therefore, CIP molecules are present as cations (CIP^+^) at pH < 5.9 because of protonation of the amine group. At pH > 8.9, CIP molecules are present as anions (CIP^−^) due to deprotonation of carboxylate. CIP molecules exist as zwitterions (CIP^0^) at pH in the range of 5.9–8.9.^33^ In this study, CIP removal efficiency increases from 80.4% to 87.5% with rising pH from 3 to 7 (Fig. 5b). The maximum removal efficiency for CIP was achieved at pH 7 which might be because of the effect of electrostatic interactions between the positive charge on the adsorbent and the negative group (–COO–) on CIP^0^. The hydrophobic interactions have also contributed because of higher hydrophobic nature of zwitterionic species between each other than its cationic and anionic form.^34,35^ Therefore, hydrophobic interactions have a considerable part in the binding interaction of CIP on F/MgO nanocomposite. The adsorption decreased to 75.2% with increasing pH to 11 because of the repulsion between anionic CIP^−^ form and the negatively charged groups on the adsorbent.

For TC, the removal percentage rose from 36.7% to 43.6% with increasing pH from 3 to 5 (Fig. 5b). Then, it decreases gradually to 33.2% at pH 11. TC is an amphoteric molecule with different ionizable functional groups that can undergo protonation–deprotonation reactions based on the aqueous solution pH. At pH < 3.3, TC is present as a cation (TC^+^), because of the protonation of dimethyl-ammonium group. When pH is between 3.3 and 7.7, TC exists as a zwitterion (TC^0^), due to the loss of a proton from the phenolic diketone moiety. At pH more than 7.7, TC is present as anion (TC^−^ or TC^2−^) due to the loss of protons from the tri-carbonyl system and phenolic diketone moiety.^36,37^ TC removal increased up to pH 5 because cationic species of TC decreased and replaced by zwitterionic, which led to a decrease in electrostatic repulsion between TC and adsorbent and enhanced the antibiotic removal. It is evident that the maximum removal of CIP and TC could be achieved at pH 7.0 and 5.0, respectively (Fig. 5b).

#### 3.3.3 Effect of adsorbent dose

The influence of adsorbent dose on removal of CIP and TC was studied in the range of 0.05–1 g L^−1^ at 30 °C with 50 mg L^−1^ of antibiotic and contact time of 60 min (Fig. 5c). For CIP, the removal percentage increased sharply from 31.1% to 84.6% with raising the adsorbent dose from 0.05 to 0.2 g L^−1^ due to availability of high surface area and adsorption sites. Thereafter, with increasing in the adsorbent dose above 0.2 g L^−1^, the CIP removal efficiency did not change greatly. That might be because of the overlapping of adsorption sites and overcrowding of adsorbent in the solution.^38,39^ Another explanation could be reaching saturation of the active sites on the surface of adsorbent. Therefore, the presence of plenty of unoccupied active sites wasn't influential in CIP removal.^40^ Therefore, 0.2 g L^−1^ of the adsorbent dose was used as the optimal dose for CIP removal in the next experiments.

TC removal efficiency increased from 4.5% to 43.7% when the adsorbent dose rose from 0.05 to 1 g L^−1^ (Fig. 5c). This could be attributed to more accessible adsorption sites on the adsorbent surface allowing more TC molecules to pass through the pores on the surface of the adsorbent.^41^ Based on our study, 0.2 g L^−1^ and 1 g L^−1^ were selected as the efficient dose for further experiments of CIP and TC removal, respectively.

#### 3.3.4 Effect of antibiotics' concentrations

The influence of initial concentrations was investigated using adsorbent dose for CIP (0.2 g L^−1^) and TC (1 g L^−1^) for 60 min at 30 °C. CIP removal percentage increased from 14.3% to 86.4%, when the initial CIP concentration increased from 10 to 60 mg L^−1^ (Fig. 5d). This might be because of increasing concentration gradients of adsorbate that formed at higher concentrations. This behavior could be related to the effect of enrichment of driving force for mass transfer between the liquid and solid phases.^42–44^ The results are in agreement with previous studies.^2,45^

As evidenced from Fig. 5d, TC adsorption efficiency decreased from 56.1% to 33.3% with increasing the TC concentration from 10 mg L^−1^ to 60 mg L^−1^. Such behavior of removal efficiency can be related to limited number of adsorption sites on the nanocomposite surface due to its fixed dose, which is saturated by increasing the TC concentration in the solution.^46^

#### 3.3.5 Effect of temperature

The energy-dependent mechanisms in the removal process have affected by the temperature of the solution. The antibiotic removal percentage was studied at temperatures from 30 °C to 50 °C (Fig. 5e). The removal efficiency for 50 mg L^−1^ CIP declined from 84.8% to 75.4% with increasing the temperature from 30 °C to 50 °C for 60 min. The decrease in CIP removal could be due to increase the movement of the CIP molecules from the adsorbent surface to the bulk phase with rising the temperature of the solution as mentioned by Mohammed *et al.*,^43^ and Zahmatkesh *et al.*^47^

In the contrary, temperature had minimal impact on removal of 50 mg L^−1^ TC for 60 min as the increase was from 43.5% to 46.8% by increasing temperature from 30 °C to 50 °C. High temperature was favorable for TC removal due to acceleration the movement of TC molecules from the solution, the improvement of adsorptive forces, or reduction in the retarding forces.^20,48^

### 3.4 Adsorption behaviors

Based on previous reports,^49,50^ the adsorption mechanism of antibiotic is managed by chemical and physical interactions. The chemical adsorption is controlled by π–π stacking interaction, electrostatic interactions and cation–π bonding. The physical adsorption is controlled by some weak intermolecular forces, such as van der Waals' force and pore-size selective.

#### 3.4.1 Thermodynamic models

The nature of the adsorption reaction was determined by calculating the change in enthalpy (Δ*H*°), entropy (Δ*S*°), and Gibbs free energy (Δ*G*°) following the method of El Bouraie *et al.*:^22^1
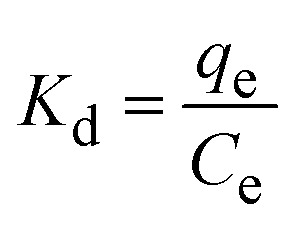

2 Δ*G*° = −*RT*ln *K*_d_

3
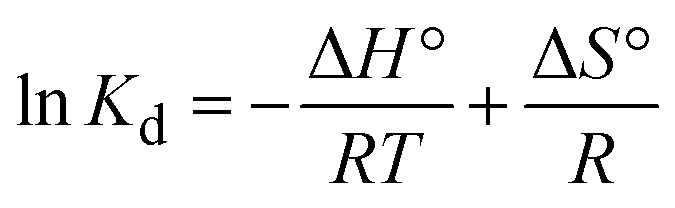
where, *k*_d_ is the distribution coefficient for adsorption (L g^−1^), *C*_e_ is concentration of antibiotic at equilibrium (mg L^−1^), *q*_e_ is the amount of antibiotic adsorbed at equilibrium per unit weight of adsorbent (mg g^−1^), *R* is the gas constant (8.31 J K^−1^ mol^−1^) and *T* is the solution temperature (K).

The thermodynamic parameters were obtained by fitting of ln *K*_d_*versus* 1/*T* (Fig. S2†). The Δ*H*° and Δ*S*° values were derived from the slope and the intercept of the Van't Hoff linear fit. Thermodynamic parameters of CIP and TC adsorption at various temperatures are summarized in Table 2.

**Table 2:** Thermodynamic parameters for CIP and TC adsorption on F/MgO nanocomposite

Antibiotic	Temperature (°C)	Δ*G*	Δ*H*	Δ*S*
CIP	30	−8.38896	−24.4	−0.053
	40	−7.73487		
	50	−7.33326		
TC	30	0.659031	5.4	0.0158
	40	0.459409		
	50	0.344364		

For CIP, all Δ*G* values were negative −8.38896, −7.73487, and −7.33326 kJ mol^−1^ at 30, 40, and 50 °C, respectively. This energy slightly increases with raising the temperature, indicating the spontaneity and feasibility of the adsorption process affirm less favorable at higher temperatures.^40^ On the other hand, all Δ*G* values for TC removal were positive being 0.659031, 0.459409, and 0.344364 kJ mol^−1^ for 30, 40, and 50 °C, respectively, suggesting that the adsorption of TC is non-spontaneous in nature.

Negative Δ*H*° value for CIP (−24.4 kJ mol^−1^) indicated that the adsorption process is exothermic, while positive Δ*H*° value for TC (5.4 kJ mol^−1^) demonstrated that the adsorption behavior is endothermic. The values of Δ*H*° confirm that active sites on the adsorbent are affected with the temperature of the reaction. If the energy required for the active sites is lower than the energy released during sorbate–sorbent interaction, the adsorption process tends to be exothermic, as observed in CIP adsorption on the nanocomposite. Therefore, the adsorption efficiency for CIP decreases with increasing temperature. Alternatively, if the energy needed for these active sites is higher than the energy released during sorbate–sorbent interaction, the interaction is likely to be endothermic, as showed in TC adsorption. Therefore, the removal efficiency of TC slightly increased with raising the temperature of the reaction. These results are in constituent with Chandrasekaran *et al.*^51^

Finally, the negative value of Δ*S*° for CIP (−0.053 J mol^−1^ K^−1^) suggest that adsorption of CIP on F/MgO nanocomposite is mainly ascribed to be physical. The positive value of Δ*S* (0.0158 J mol^−1^ K^−1^) demonstrated that the adsorbed nature of TC was random and there was clear affinity between TC and the surface of the adsorbent. In summary, the adsorption process of CIP is spontaneous, exothermic, and mainly physical. Conversely, the adsorption process of TC on the F/MgO nanocomposite is non-spontaneous, endothermic, and random while is attributed to chemical adsorption.

#### 3.4.2 Kinetic isotherm models

Kinetic models of the studied antibiotics were calculated to determine the mechanisms involved. The pseudo-first order and pseudo-second order kinetic models, and intraparticle diffusion model are expressed by the following equations:^4,52^

4 log(*q*_e_ − *q*_*t*_) = log *q*_e_ − *K*_1_*t*/2.303

5 *t*/*q*_*t*_ = 1/*K*_2_*q*_e_^2^ + *t*/*q*_e_

6 *q*_*t*_ = *K*_diff_*t*^0.5^ + *L*

where *K*_1_ is the first-order rate constant (min^−1^), *K*_2_ is the second-order rate constant (g mg^−1^ min^−1^), *q*_*t*_ (mg g^−1^) is the adsorption capacity of antibiotic adsorbed on nanocomposite at time *t* (min), *K*_diff_ is the intraparticle diffusion constant (mg g^−1^ min^−0.5^), and *L* represents the layer thickness (g mg^−1^).

Data of kinetics models and correlation coefficients are shown in Fig. S3† and Table 3. The fitting of the pseudo-first order model for the studied antibiotics failed because the calculated values of capacity (*q*_e,cal_) were small 55.7 and 1.3 mg g^−1^ for CIP and TC, respectively, comparing to the values of the experimental (*q*_e,exp_) 217.8 and 21.0 mg g^−1^ for CIP and TC, respectively. The values of *q*_e,cal_ of the Pseudo-second order model were 222.2 mg g^−1^ for CIP and 21.3 mg g^−1^ for TC which were similar to the values of *q*_e,exp_ (217.8 mg g^−1^ for CIP and 21.0 mg g^−1^ for TC). Moreover, the correlation coefficients (*R*^2^) for two antibiotics are more suitably fitted by the pseudo-second order model due to their higher values (*R*^2^ are 0.99 for both antibiotics) than the values of pseudo-first order model (0.91 for CIP and *R*^2^ is 0.47 for TC). Thus, the pseudo-second order model was more suitable in describing the adsorption kinetics of CIP and TC on the F/MgO nanocomposite. The kinetics constant *K*_2_ for CIP and TC were 0.002 and 0.02 g mg^−1^ min^−1^, respectively. The mentioned results in our study are confirming that the adsorption mechanism could be depending on chemical interactions that include electron transfer and electron sharing between adsorbate and adsorbent.

**Table 3:** Kinetics parameters for the adsorption of TC and CIP on nanocomposite

Pseudo-first order model	Antibiotic	*q*_e,exp_	*K*_1_	*q*_e,cal_	*R* ^2^
	CIP	217.8	0.06	55.7	0.91
	TC	21	0.03	1.3	0.47
Pseudo-second order model	Antibiotic	*q*_e,exp_	*K*_2_	*q*_e,cal_	*R* ^2^
	CIP	217.8	0.002	222.2	0.99
	TC	21	0.02	21.3	0.99
Intraparticle diffusion	Antibiotic	*K*_diff_		*R* ^2^	
	CIP	4.478		0.61	
	TC	0.384		0.51	

Additionally, the intraparticle diffusion model (Fig. S3† and Table 3) presents detailed insights of the mass transfer process of antibiotic adsorption on F/MgO nanocomposite. Plots (Fig. S3†) are segmented into three stages for CIP and TC. The initial stage shows rapid adsorption of both antibiotics, driven by the diffusion of antibiotic molecules from the solution to the outer adsorbent surface. The second stage represents a slower adsorption stage, which is a rate-limiting step. Finally, the third stage corresponds to the equilibrium phase. However, lines do not pass through the origin, suggesting that intra-particle diffusion is not the sole rate-controlling step in the overall adsorption process.^4^

A summary of previous literature examined antibiotic removal using other adsorbents is presented in Table 4. It is evident that F/MgO nanocomposite achieved high antibiotic removal compared with another adsorbent. Further studies are recommended to investigate F/MgO nanocomposite in removal other organic pollutants.

**Table 4:** Comparison of maximum adsorption capacity of CIP and TC with previously studies

Adsorbent	Antibiotic	*q*_m_ (mg g^−1^)	pH	Dose (g L^−1^)	Ref.
BC-NiS	CIP	41.66	—	2.0	53
BC-NiFe_2_O_4_		68.79	—	2.0	4
Co-doped UiO-66		45.17	5.0	0.3	40
F/MgO		217.8	7.0	0.2	This study
BNNSs	TC	346.66	8.0	0.2	19
BC-700		11.9	7.0	3.2	41
ZIF-8		303.0	4.0	0.5	46
F/MgO		21.0	5.0	1.0	This study

#### 3.4.3 Adsorption activity

The adsorption performance was investigated using UV-VIS spectroscopy and FTIR for 50 mg L^−1^ of CIP and TC before and after adsorption on the surface of the F/MgO nanocomposite at the optimized conditions, for CIP (pH 7, 0.2 g L^−1^ of F/MgO) and TC (pH 5, 1 g L^−1^ of F/MgO) for 60 min at 30 °C.

**Fig. 6:**
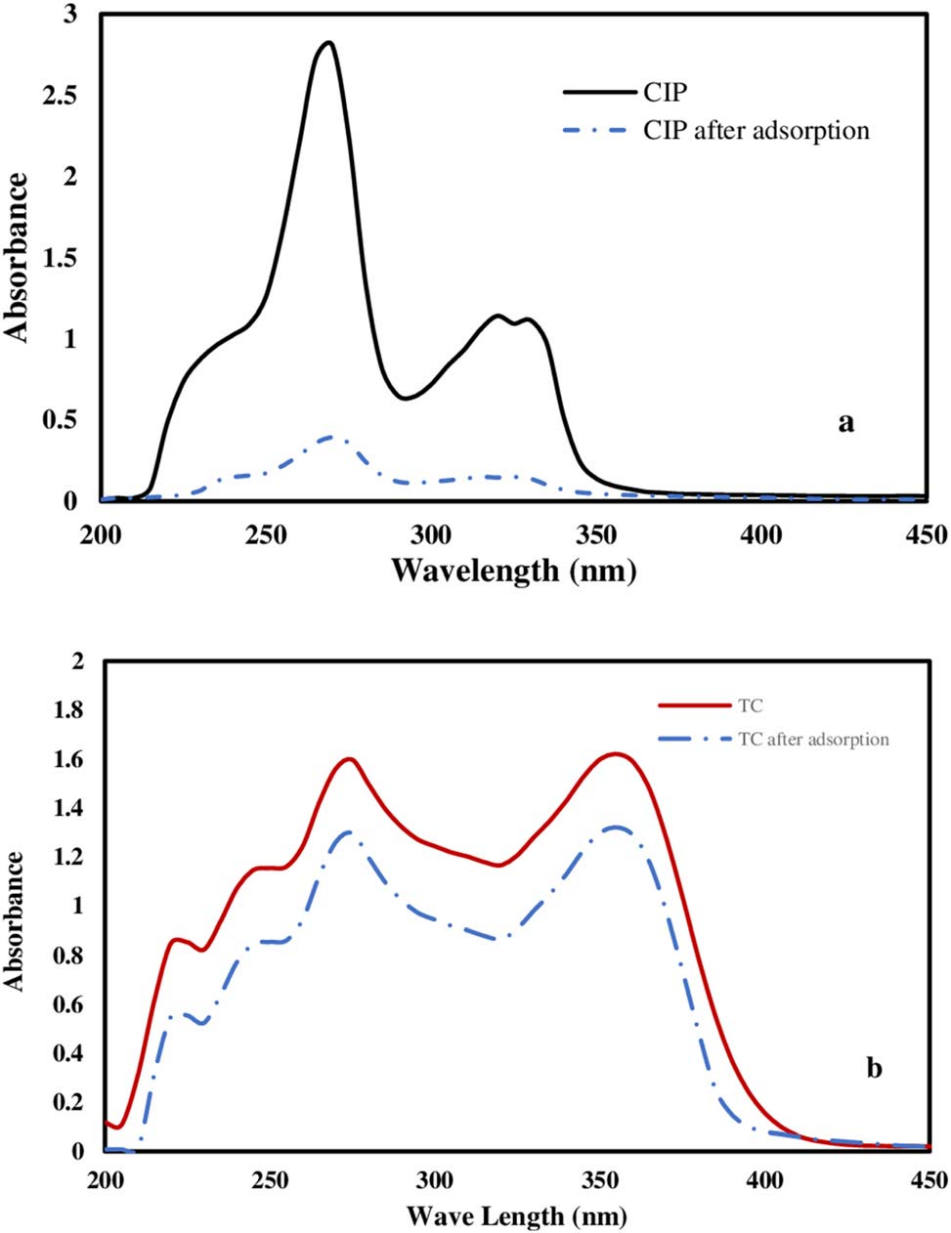
UV–Vis absorption spectrum before and after the adsorption process of 50 mg L^−1^ CIP solution (a), and TC solution (b) at the optimized conditions for 60 min at 30 °C.

Fig. 6 presents the UV–VIS spectra of CIP and TC before and after adsorption on the surface of the F/MgO nanocomposite at the optimized conditions for each antibiotic. For CIP (Fig. 6a), the position of peaks did not change but their intensity decreased after adsorption. In case of TC, there are two chromophoric regions which localized on ring A and ring BCD of TC molecule (Fig. S4†). These chromophoric regions are responsible for the pale to bright yellow color of TC. The peak at 275 nm is attributed to π = π* transition of the tricarbonyl system of ring A. All bands produced by π = π* transition of ketophenolic hydroxyl groups of the BCD chromophore. These transitions are sensitive to deprotonation and metallic coordination.^54,55^ As shown in Fig. 6b, the intensity of TC bands at 358 and 275 nm decreased after adsorption on F/MgO nanocomposite.

**Fig. 7:**
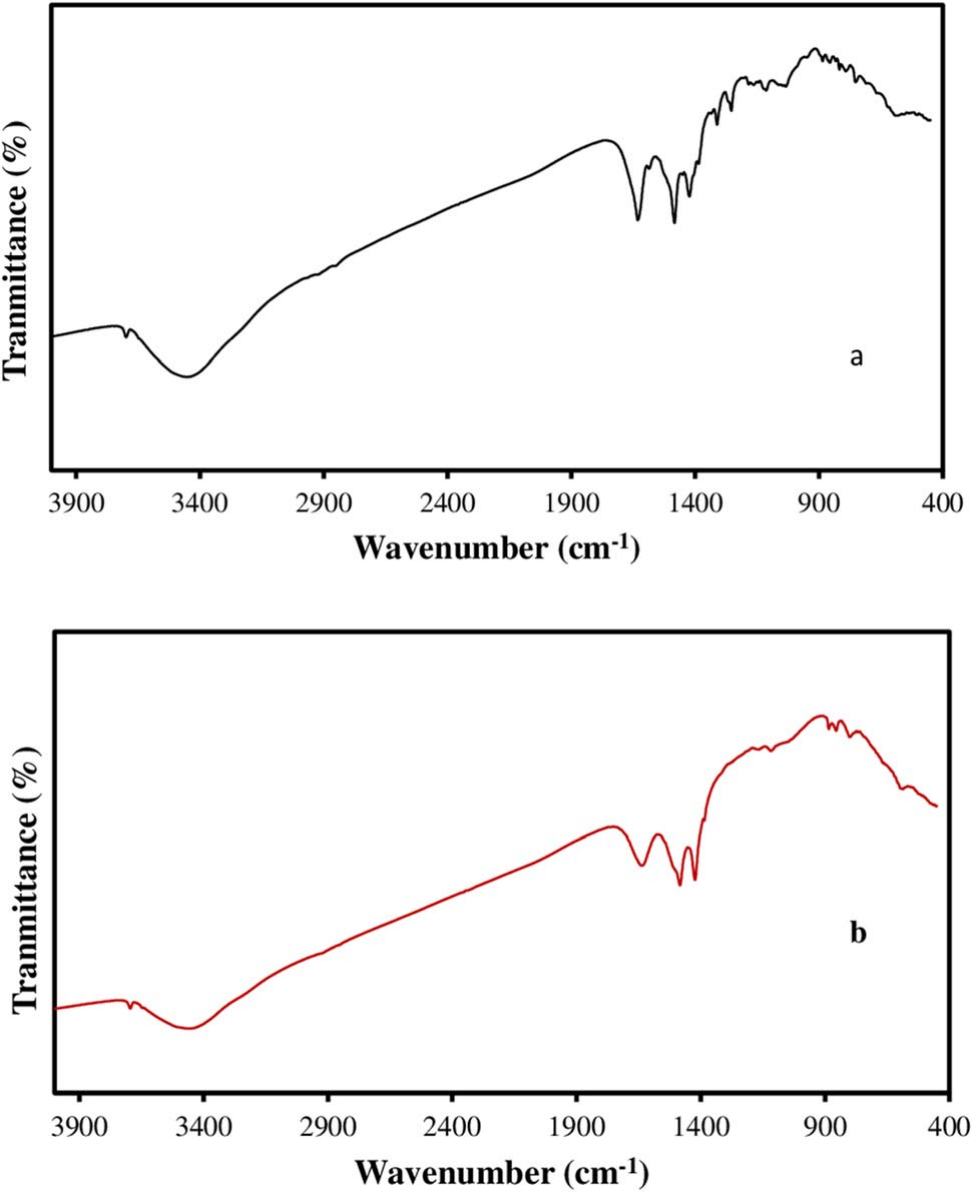
FTIR after the adsorption process of CIP (a), and of TC (b).

FTIR were studied after adsorption of CIP and TC on F/MgO nanocomposite (Fig. 7) comparing with Fig. 2b. The same pattern of F/MgO nanocomposite presented in Fig. 7a and b which verified the stability of the F/MgO nanocomposites after adsorption process. The changes in density of different adsorption bands of F/MgO nanocomposite were observed (Fig. 7a and b). There are significant differences in the density of absorption peaks of C=C, C=O, and O–H cm^−1^. These results confirm that there are interactions between the adsorbent and antibiotic molecules.

### 3.5 Effect of ionic strength on antibiotics' removal

**Fig. 8:**
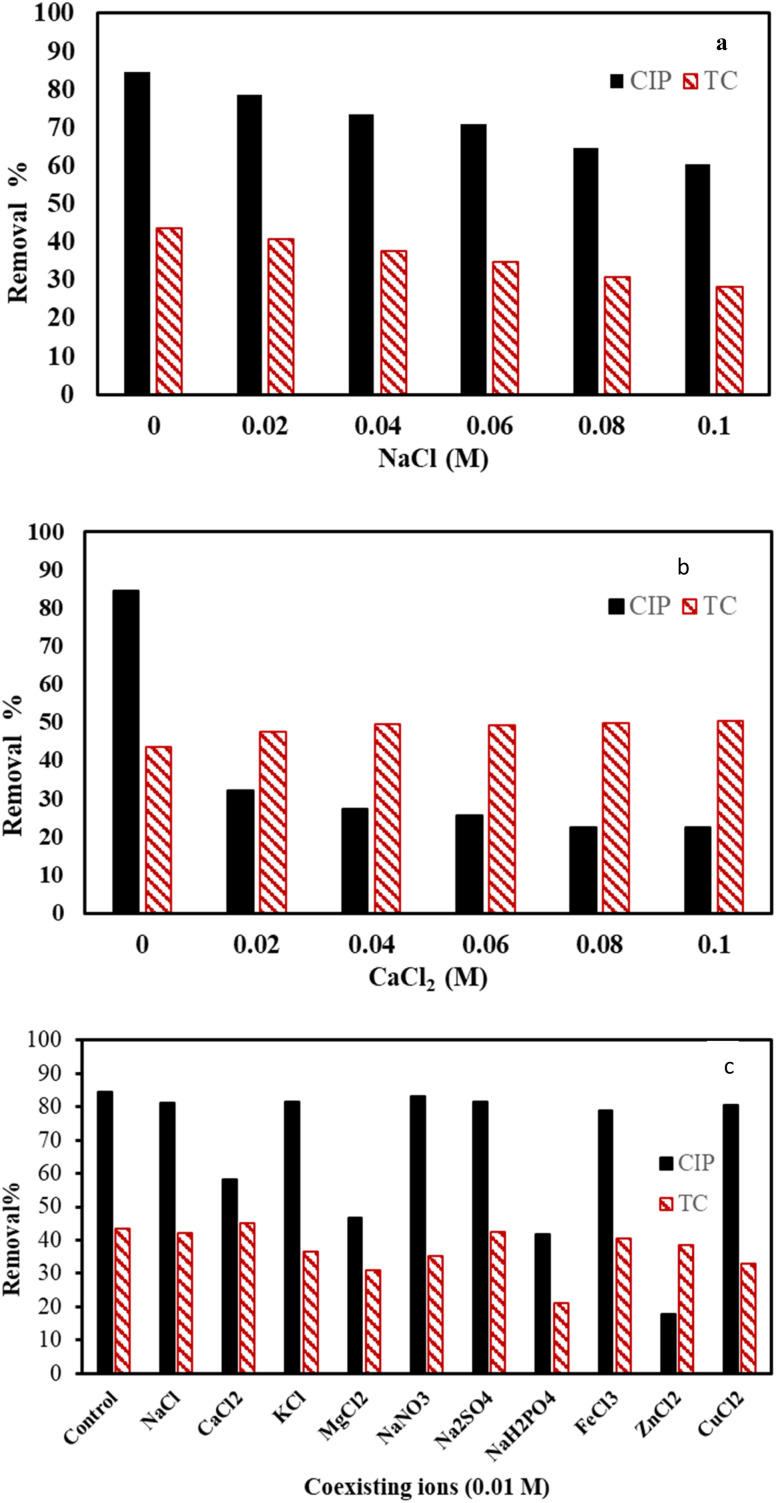
Effect of NaCl (a), CaCl_2_ (b), different ions (c) on CIP and TC removal.

The presence of different ions in domestic water and industrial wastewater may influence the adsorption process of organic contaminants.^20^ Therefore, we examined the impact of some ions on the adsorption process of 50 mg L^−1^ of CIP and TC using the F/MgO nanocomposite (0.2 g L^−1^ for CIP and 1 g L^−1^ for TC) for 60 min at 30 °C. In Fig. 8a, NaCl caused a negative impact on antibiotic removal. When NaCl concentrations incrementally increased to 0.1 M, the removal efficiency for CIP and TC declined from 84.6% to 60.4%, and from 43.6% to 28.2%, respectively. This decline may be ascribed to the interactions of Na^1+^ and Cl^1−^ ions with zwitterionic antibiotic molecules in the solutions, which can hinder the contact between the antibiotic and adsorbent.^56^

Additionally, with increasing the concentration of CaCl_2_ from 0 to 0.1 M, CIP removal declined from 84.6 to 22.5% (Fig. 8b). On the contrary, the removal efficiency of TC increased from 43.6% to 50.3% with raising the concentration of CaCl_2_ from 0 to 0.1 M. Calcium ions act as bridge between the adsorbate and adsorbent. They react with oxygen-containing functional groups (as –OH and –C=O) of TC molecule and form strong insoluble complex that enhance the adsorption of TC. The TC–Ca complexes boosted the new adsorption active sites for further removal of TC. This explanation is also confirmed by Xiang *et al.*, 2020.^57^ On the contrary, other studies presented that existence of Ca^2+^ ions inhibited the adsorption of TC on the adsorbent.^46,58,59^

As wastewaters have different types of salts; the effect of coexisting ions should be considered.^46^Fig. 8c presents the influence of various ions on the adsorption of TC and CIP. Results indicate that K^1+^ has less effect on the removal of CIP than Ca^2+^ (Fig. 8c). This might be attributed to two factors; firstly, Ca^2+^ ions have higher valence than K^1+^, which increase the binding on the adsorbent surfaces. Secondly, Ca^2+^ ion can occupy two active sites on the adsorbent surfaces, while K^1+^ ion binds to one interaction site.^60,61^ Removal for CIP and TC were mainly decreased in presence of different co-existing salts, except presence of Ca^2+^ for TC removal, comparing with control. This might be due to different affinity of those ions to the active sites on the adsorbent.^62,63^ Mg and H_2_PO_4_ exerted strong inhibitory effects on both TC and CIP adsorption because Mg has the highest atomic radius and valence in the used cations.

### 3.6 Adsorption of antibiotic in the binary system

**Fig. 9:**
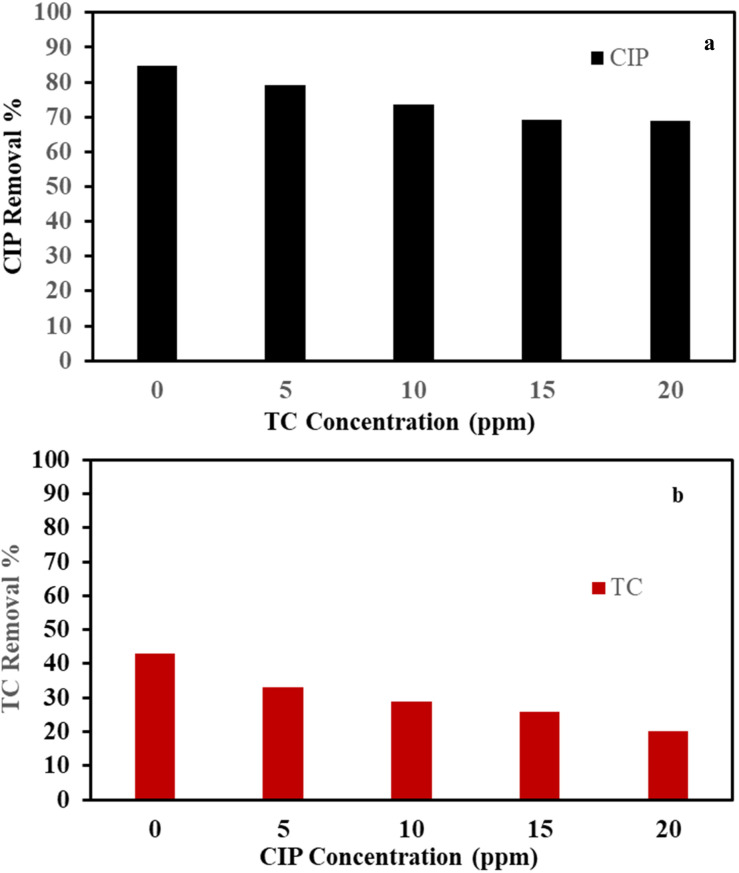
Removal of CIP in presence of 0–20 mg L^−1^ TC (a) removal of TC in presence of 0–20 mg L^−1^ CIP (b).

The adsorption of CIP and TC in binary mixtures was competitive to each other because of the adsorption affinity of CIP or TC on the surface of the adsorbent. The removal of 50 mg L^−1^ of CIP or TC in binary mixtures at different concentrations (0–20 mg L^−1^) of another antibiotic using optimized dose of F/MgO nanocomposite (0.2 for CIP and 1 g L^−1^ for TC) for 60 min at 30 °C are presented in Fig. 9. With increasing TC concentrations from 0 to 20 mg L^−1^, the adsorption of CIP decreased from 84.6 to 68.9%. TC removal declined from 43.0 to 20.3% using CIP concentrations from 0 to 20 mg L^−1^. Our results showed that there is competitive sorption between CIP and TC for the limited sites on the adsorbent.

### 3.7 Environmental application of F/MgO nanocomposite for CIP and TC removal

Two samples were collected from tap water and river water (Alexandria, Egypt). The samples did not contain CIP and TC. 50 mg L^−1^ of CIP or TC was spiked to each water sample. Then, the adsorption process was conducted to those samples using optimized dose of F/MgO nanocomposite for CIP (0.2 g L^−1^) or for TC (1 g L^−1^) without pH control for 60 min at 30 °C. As shown in Table 5, the removal efficiency for CIP and TC lower than the removal from distilled water sample. This decrease in removal percentage of antibiotic in water sources samples can be explained by the presence of interfering ions.

**Table 5:** The removal percentage of CIP and TC using F/MgO nanocomposite from different water sources

Antibiotic	Distilled water	Tap water	River water
CIP	84.5	81.9	76.6
TC	43.4	40.1	38.7

### 3.8 Reusability of the adsorbent

**Fig. 10:**
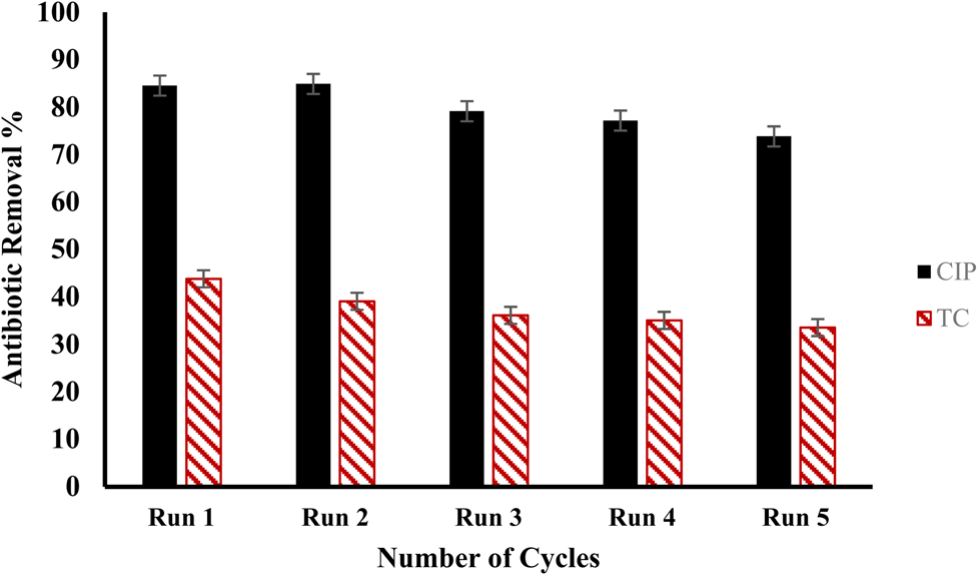
The reusability of F/MgO nanocomposite for removal of CIP and TC.

The reutilization of adsorbents has cost-benefit for wastewater treatment process. Highly reusable adsorbents contribute in operational costs reduction and stability during their usage.^64^ Results of five cyclic adsorptions of 50 mg L^−1^ CIP and TC by optimal doses of F/MgO nanocomposite for 60 min at 30 °C are presented in Fig. 10. The removal percentage of CIP and TC decreased after five successive runs from 84.5% to 73.8%, and from 43.7% to 33.5%, respectively. F/MgO nanocomposite retained good removal for antibiotics after five cycles. This confirmed that F/MgO nanocomposite has remarkable adsorbability, reusability, and potential for practical applications.

## 4. Conclusion

In conclusion, a new F/MgO nanocomposite was prepared and explored for CIP and TC removal in aqueous solutions. The highest removal efficiency at a dose of 50 mg L^−1^ CIP was 84.6% under the conditions of 60 min, pH 7, and an adsorbent dose of 0.2 g L^−1^. For 50 mg L^−1^ of TC, the maximal removal was 43.6% at 60 min, pH 5, and 1 g L^−1^ of an adsorbent dose. The adsorption performances were investigated, involving adsorption thermodynamics, kinetics, UV/VIS scanning, and FTIR. Based on thermodynamic models, the adsorption of CIP was spontaneous, exothermic, and is mainly physical. TC adsorption process was non-spontaneous, endothermic, and ascribed to chemical adsorption. Adsorption data for CIP and TC fitted well with the pseudo-second-order kinetic models influenced by chemical interactions. Existence of different coexisting ions affected on the adsorption process of CIP and TC. The removal efficiency for CIP and TC in the binary system were explored. This work introduced a novel adsorbent for elimination of CIP and TC from aqueous solutions.

## Data availability

The data supporting this article have been included as part of the ESI.†

## Author contributions

Sammer M. Bekhit performed experiments, analyzed data, and wrote the manuscript. All authors contributed in the design of experiments, discussion results, and revising of the manuscript.

## Conflicts of interest

There are no conflicts to declare.

## Supplementary Material

RA-015-D4RA07938H-s001
