# Supplementary material for: Effectiveness of fullerene/magnesium oxide nanocomposite in removing ciprofloxacin and tetracycline from aqueous solutions
Source: RSC Adv. 2025 Feb 17;15(7):5190–201. doi: 10.1039/d4ra07938h (PMC11831255; doi:10.1039/d4ra07938h)
Supplement: RA-015-D4RA07938H-s001 [file RA-015-D4RA07938H-s001.pdf]

Supplementary Information (SI) for RSC Advances.
This journal is © The Royal Society of Chemistry 2025

Preparation of MgO NFs

Briefly, 10 g of $MgCl_2$ anhydrous was dissolved in 100 ml of distilled water, and ammonia solution (1 N) was added drop-wise at 50 ℃ under vigorous stirring till the solution pH reached 10. The resulting white precipitate of $Mg(OH)_2$ was centrifuged for 5 min at 3000 rpm. Subsequently, it was washed with distilled water three times and then dried overnight at 110 ℃. The dried powder was finally calcined at 500 ℃ in a muffle furnace for 3 h to obtain MgO NFs.

Text 1

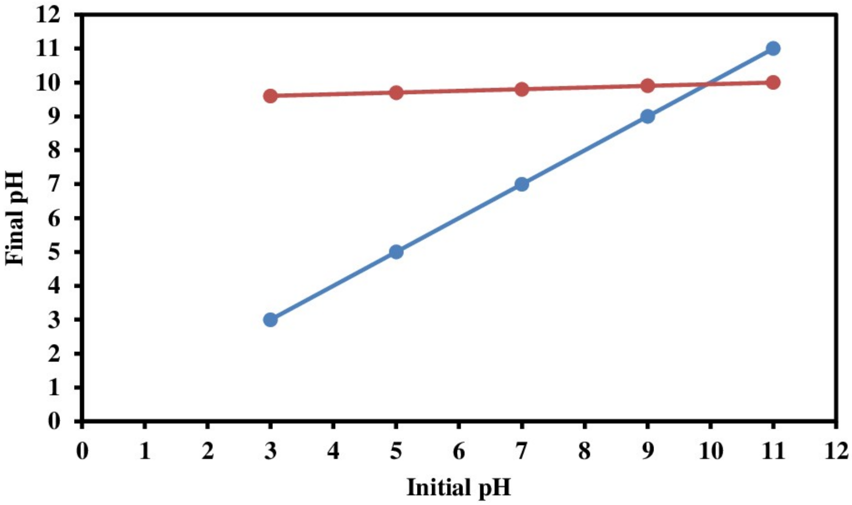

Fig. S1: Zeta Potential of F/MgO Nanocomposite at Different pH

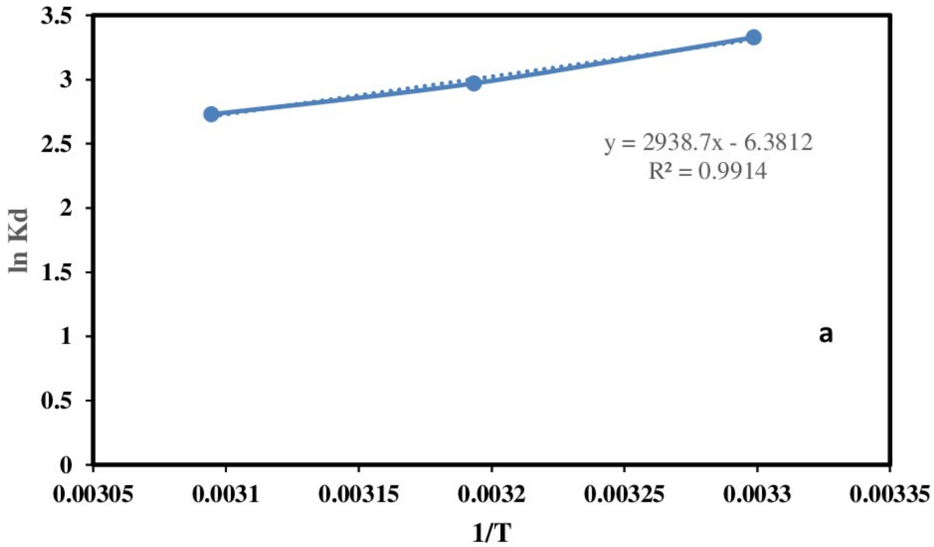

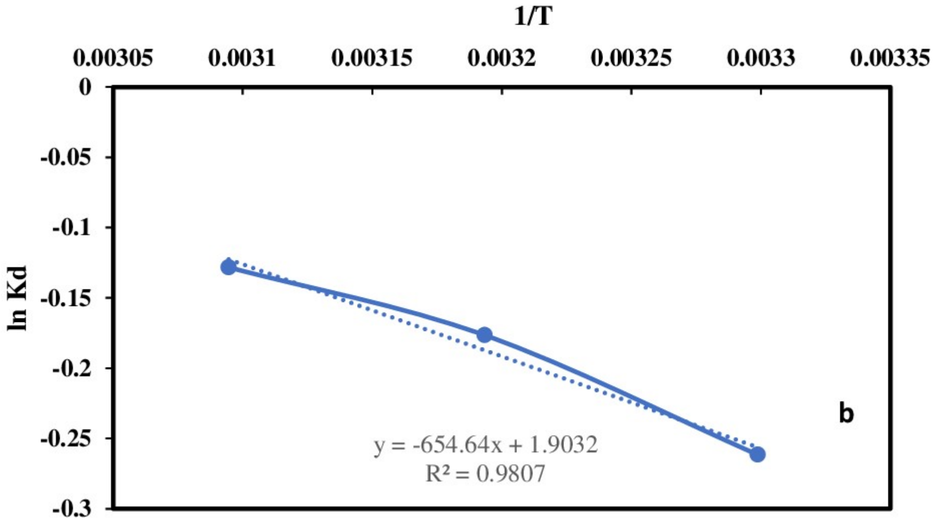

Fig. S2: Thermodynamic Model for CIP (a), TC (b)

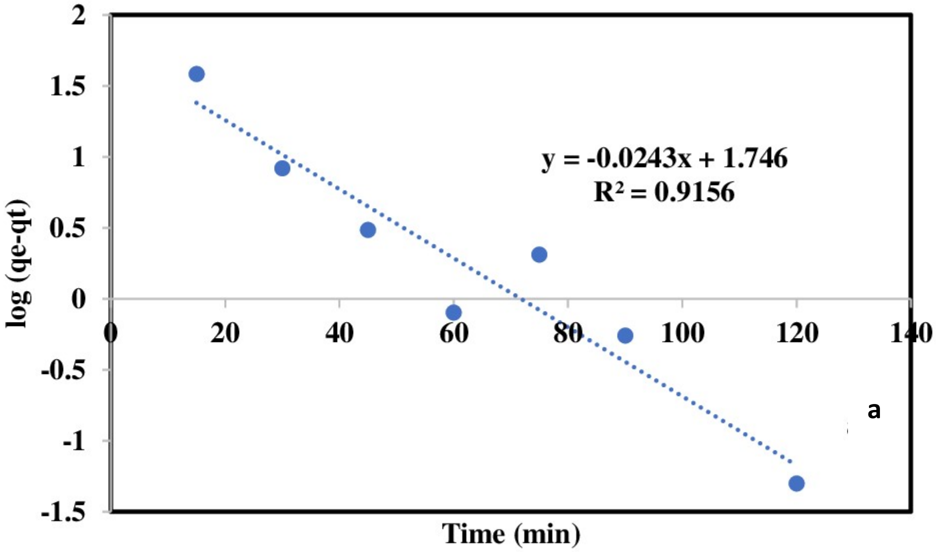

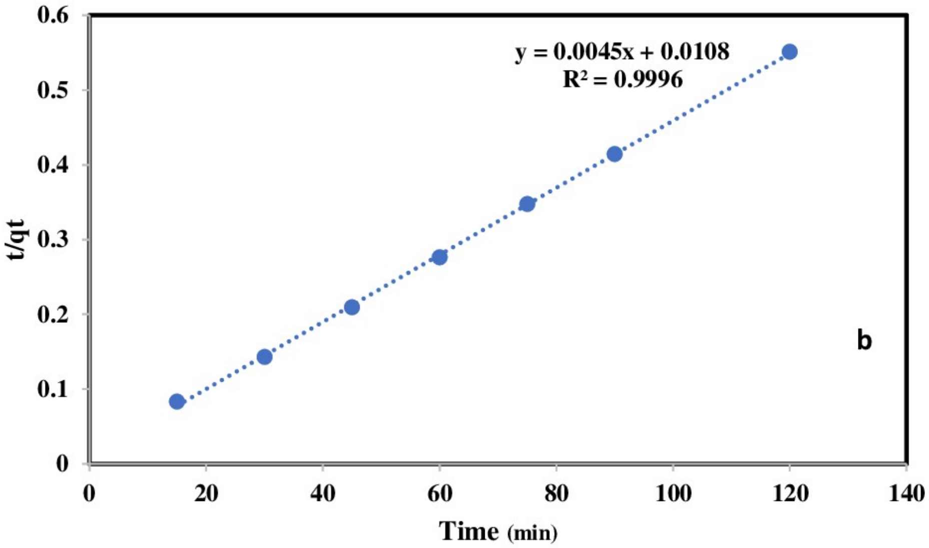
y = 0.0045x + 0.0108
R² = 0.9996
b
t/qt
Time (min)
0
0.1
0.2
0.3
0.4
0.5
0.6
0
20
40
60
80
100
120
140

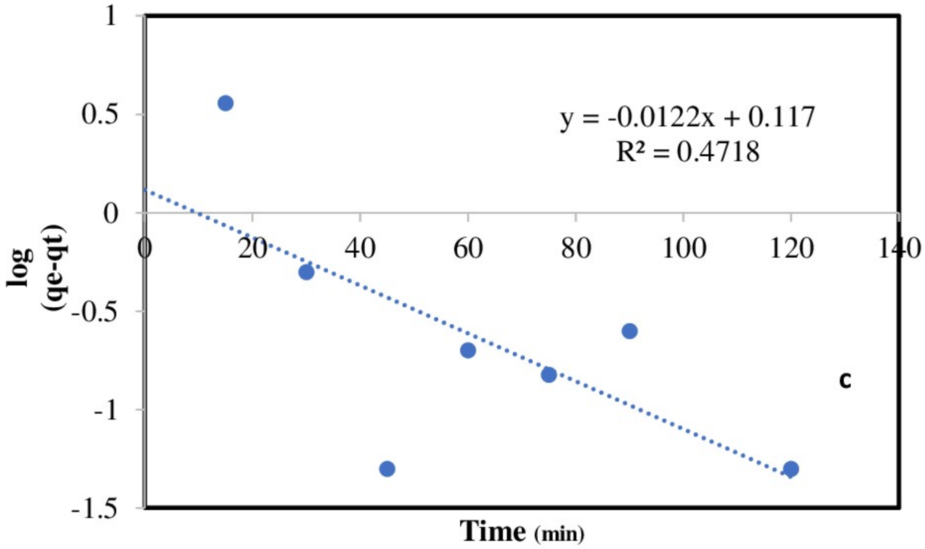
y = -0.0122x + 0.117
R² = 0.4718
c
log (qe-qt)
Time (min)
1
0.5
0
-0.5
-1
-1.5
0
20
40
60
80
100
120
140

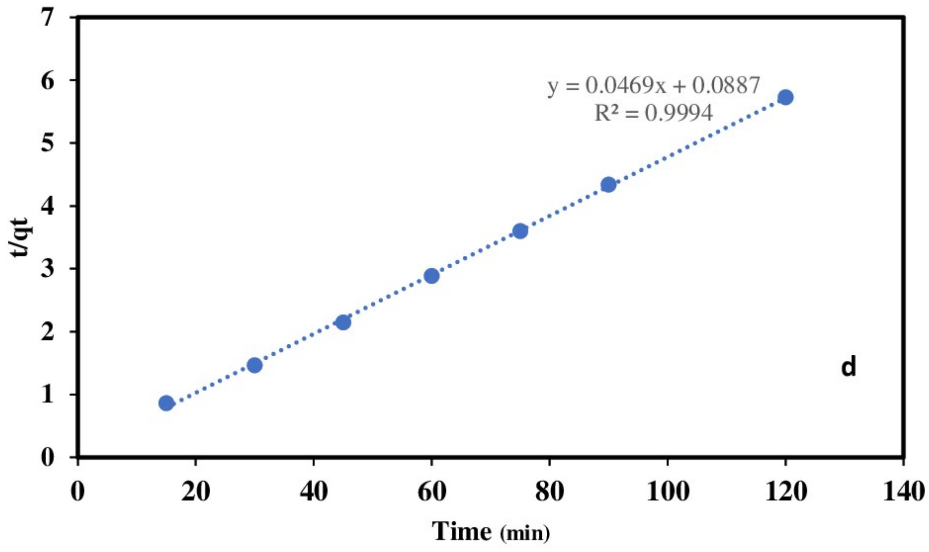

y = 0.0469x + 0.0887
R² = 0.9994
d
t/qt
Time (min)
0
1
2
3
4
5
6
7
0
20
40
60
80
100
120
140

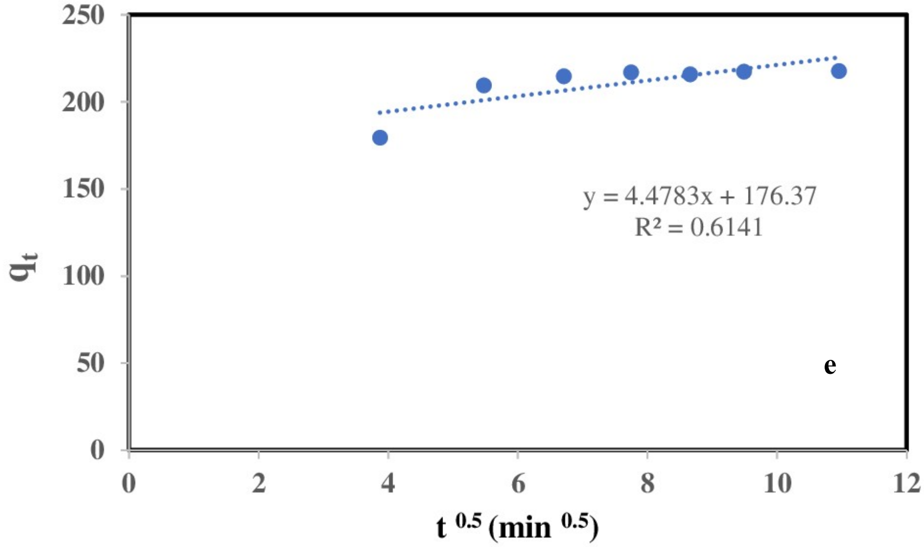

y = 4.4783x + 176.37
R² = 0.6141
e
q_t
t 0.5 (min 0.5)
0
50
100
150
200
250
0
2
4
6
8
10
12

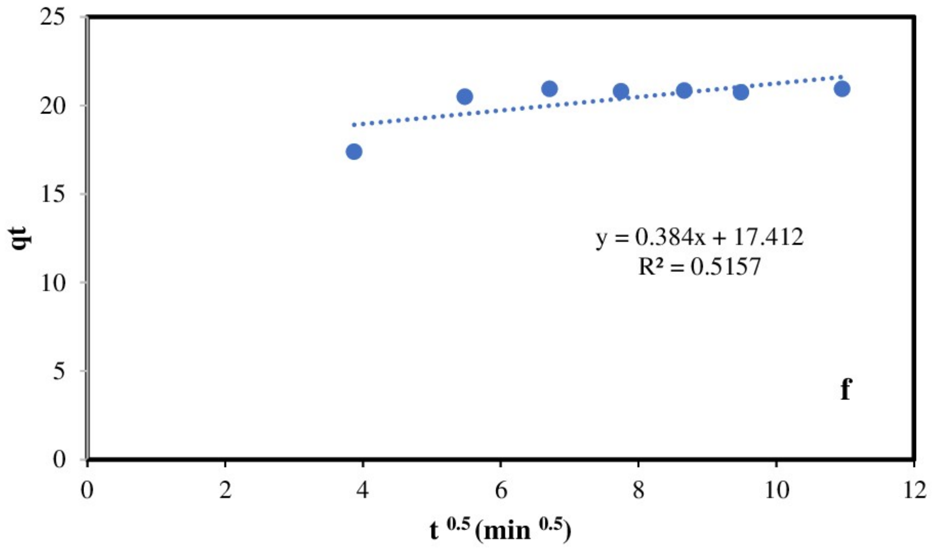

Fig. S3: Pseudo-first order Model (a), Pseudo-second order Model of CIP on F/MgO (b), Pseudo-first order Model (c), Pseudo-second order Model of TC on F/MgO (d), Intraparticle diffusion model of CIP (e), of TC (f)

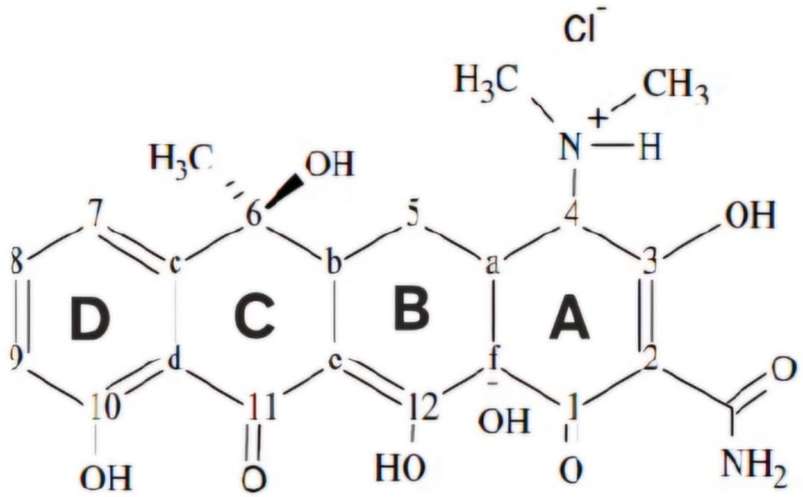

Fig. S4: Molecular Structure of Tetracycline Hydrochloride
